# Connect With Me. Exploring Influencing Factors in a Human-Technology Relationship Based on Regular Chatbot Use

**DOI:** 10.3389/fdgth.2021.689999

**Published:** 2021-11-18

**Authors:** Lara Christoforakos, Nina Feicht, Simone Hinkofer, Annalena Löscher, Sonja F. Schlegl, Sarah Diefenbach

**Affiliations:** Department of Psychology, Ludwig-Maximilians-Universität München, Munich, Germany

**Keywords:** human-computer interaction, human-technology relationship, social connectedness, anthropomorphism, social presence, digital health technologies, conversational chatbot

## Abstract

Companion technologies, such as social robots and conversational chatbots, take increasing responsibility for daily tasks and support our physical and mental health. Especially in the domain of healthcare, where technologies are often applied for long-term use, our experience with and relationship to such technologies become ever more relevant. Based on a 2-week interaction period with a conversational chatbot, our study (*N* = 58) explores the relationship between humans and technology. In particular, our study focuses on felt social connectedness of participants to the technology, possibly related characteristics of technology and users (e.g., individual tendency to anthropomorphize, individual need to belong), as well as possibly affected outcome variables (e.g., desire to socialize with other humans). The participants filled in short daily and 3 weekly questionnaires. Results showed that interaction duration and intensity positively predicted social connectedness to the chatbot. Thereby, perceiving the chatbot as anthropomorphic mediated the interrelation of interaction intensity and social connectedness to the chatbot. Also, the perceived social presence of the chatbot mediated the relationship between interaction duration as well as interaction intensity and social connectedness to the chatbot. Characteristics of the user did not affect the interrelations of chatbot interaction duration or intensity and perceived anthropomorphism or social presence. Furthermore, we did not find a negative correlation between felt social connectedness of users to the technology and their desire to socialize with other humans. In sum, our findings provide both theoretical and practical contributions. Our study suggests that regular interaction with a technology can foster feelings of social connectedness, implying transferability of dynamics known from interpersonal interaction. Moreover, social connectedness could be supported by technology design that facilitates perceptions of anthropomorphism and social presence. While such means could help to establish an intense relationship between users and technology and long-term engagement, the contexts in which anthropomorphic design is, actually, the means of choice should be carefully reflected. Future research should examine individual and societal consequences to foster responsible technology development in healthcare and beyond.

## Introduction

Companion technologies increasingly become a part of our everyday lives and assist us in our household, shopping, and other tasks. Especially in the domain of healthcare, companion technologies such as social robots and conversational chatbots play an important role and are often implemented to support physical and mental health [e.g., (1, 2)]. Therefore, within this field, the subjective user experience (UX) and personal relationship of users to such technologies seem essential. Recent research in this regard has, for example, focused on how chatbots providing online medical advice should interact with users. Results showed that expression of sympathy and empathy was favored over unemotional provision of advice (1). Furthermore, De Gennaro et al. (2) found that the participants who interacted with an empathetic chatbot reported more positive mood than the participants whose reactions were merely acknowledged by the chatbot. Such studies typically focus on single short-time interactions between human and technology or resulting UX variables, respectively.

Yet, relationships are typically not characterized by one-time experiences. According to Hinde (3), they involve multiple interactions between two individuals, which are known to each other. Based on previous research indicating that humans apply social rules from interpersonal interaction to interaction with non-human agents [e.g., (4)], this can also apply for human-technology relationships. Therefore, studies with a single session of interaction between users and technology only provide a small snapshot of a possible human-technology relationship for the exploration of its nature as well as potential influencing factors. Additionally, according to several longitudinal studies with social robots (5, 6), as users become more familiar with technologies, their perceptions of social affordances can adapt (7). Especially, in the domain of healthcare, technologies are often applied for long-term use with the goal of representing a sort of companion technology. Thus, particularly within this domain, it appears advantageous to consider possible influencing factors of a human-technology relationship based on regular interaction over a certain period of time. Furthermore, recent research has suggested a possible influence of anthropomorphism and social presence as characteristics of a technology, which could play a role for felt social connectedness of users to the technology. Kang and Kim (8), for example, found that anthropomorphism resulted in more positive user responses by increasing the sense of connectedness within an interaction between a human and smart objects. Similarly, the perception of social presence in a technology appears to come with the potential to provoke social responses (9), which are core to the development of connectedness to the technology (8, 10). Moreover, although social connectedness to a technology appears to positively influence various UX variables (8), from a societal perspective, it seems important to further highlight possible effects on the desire of users to socialize with other humans. According to Krämer et al. (11), for example, the participants with a high need to belong reported lower willingness to engage in social activities after interacting with a virtual agent, when the agent showed socially responsive behavior.

Our research aims at exploring the relationship between humans and technology. Within the context of a regular human-technology interaction over a 2-week period, we focus on the social connectedness to a technology as a central determinant of a human-technology relationship (12). We further explored characteristics of the technology as well as the user, which could play a role in this interrelation, including possible effects on the desire of a user to socialize with other humans.

Results of our research could contribute to human-computer interaction (HCI) research in general through insights into the nature of the relationship between humans and technology as well as influencing factors in this regard. Our study further extends existing research by considering factors of long-term use. Additionally, results regarding effects on interpersonal relationships of users could allow a more reflected and responsible use of the technologies in question, especially since, in healthcare, their use should benefit the health of users. For practice, insights into specific design elements that affect perception of users of social connectedness to a technology could be derived.

In the following sections, we outlined theoretical and empirical work on the human-technology relationship, relevant characteristics of technology and users in this relationship, as well as possible effects on interpersonal interaction, from which we derive our research hypotheses. We presented our study paradigm, methods, and results, followed by their discussion, including methodological and contextual limitations as well as implications as a basis to suggest directions for future research.

## Human-Technology Relationship

According to the “computers are social actors” (CASA) paradigm (13), individuals apply social rules from interpersonal interaction to interaction with non-human agents (4, 14). In line with this, various HCI and human-robot interaction (HRI) studies suggest that humans tend to form and maintain relationships with non-human agents (15–20). Kim et al. (19), for example, could show that the perceived benefit of being in a relationship with a robot mediated the effect of the caregiving role of the robot on relationship satisfaction of users.

A central determinant of perceived companionship as a form of aspired relationship between users and technology, especially in the domain of healthcare [e.g., (21)], seems to be social connectedness (12). With regard to interpersonal relationships, Van Bel et al. (10) describe social connectedness as an experience of belonging and relatedness, which is based on quantitative and qualitative social evaluations as well as relationship salience. In line with the assumed transferability of interpersonal dynamics to HCI [e.g., (4)], literature on consumer psychology implies that individuals can invest their feelings, values, and identities in digital possessions similar to physical ones (22, 23). According to Clayton et al. (24), this can lead to a strong sense of connectedness to such digital possessions. Kang and Kim (8) further support the role of perceived connectedness to a technology as a determinant of the human-technology relationship. They found that, by increasing a sense of connectedness, anthropomorphism of the technology comes with more positive user responses, such as a more positive attitude toward the technology or an increased intention to learn from it (8).

### Antecedents of Social Connectedness to a Technology

Regarding possible antecedents of social connectedness to a technology, previous studies have focused on recent interaction and awareness information (25). Theoretical work on the development of interpersonal relationships implies that social penetration, achieved through self-disclosure as a process of revealing information about oneself (26), is crucial to the development of interpersonal relationships (27, 28). Accordingly, the intensity of information exchange influences the development of interpersonal relationships. In this regard, two central factors are breadth and depth of information exchange. The former refers to the number of various topics discussed, whereas the latter refers to the degree of intimacy that accompanies the interactions in question (27, 28). Furthermore, Granovetter (29) describes the “strength” of interpersonal ties to be a “combination of the amount of time, the emotional intensity, the intimacy (mutual confiding), and the reciprocal services, which characterize the tie” [(29) p. 1361]. In analogy, the time spent interacting with a conversational technology as well as the perceived intensity of interaction could foster the development of a human-technology relationship, i.e., social connectedness of users to the chatbot. Thus, we hypothesize the following:

H1: The higher the interaction duration, the higher the social connectedness to the chatbot.

H2: The higher the interaction intensity, the higher the social connectedness to the chatbot.

### Effects of Technology and User Characteristics on Human-Technology Relationship

According to literature, further factors influencing the social connectedness of the user to the technology could be characteristics of the technology such as anthropomorphism and social presence. Anthropomorphism refers to the attribution of humanlike physical features, motivations, behaviors, emotions, and mental states to non-human agents or objects (30, 31). Kang and Kim (8), for example, have found that anthropomorphism increases the sense of connectedness between users and technology, which, in turn, elicits more positive user responses. Furthermore, in line with the CASA paradigm (4, 13), study results [e.g., (32, 33)] support that anthropomorphic design cues, e.g., humanlike agents on technology interfaces, lead users to perceive the interaction with the technology as more social and interpersonal.

Social presence stands for a mental simulation of other intelligences (34). According to Lee (35), in the context of HCI, social presence represents a “psychological state in which virtual social actors are experienced as actual social actors in either sensory or non-sensory ways” [(35) p. 27]. Accordingly, users do not perceive artificiality or para-authenticity in the respective technology and respond to it as if it were human (35). Moreover, earlier research has shown that social responses of individuals to computers and artificial actors were mediated by the perception of social presence during an HCI (36). Furthermore, Lee et al. (9) found that the perception of social presence of an agent mediated evaluation of participants of such. Similarly, Kim et al. (19) showed that the feeling of social presence regarding a robot had a significant positive effect on the evaluation of the robot regarding relationship satisfaction or attachment. The perception of anthropomorphism or social presence in a conversational chatbot could thus affect how users perceive their relationship to the chatbot and, therefore, how socially connected they feel to such. Consequently, we hypothesize the following:

H3: The relationship of interaction duration and social connectedness to the chatbot is mediated through

(a) perceived anthropomorphism of the chatbot.

(b) perceived social presence of the chatbot.

H4: The relationship of interaction intensity and social connectedness to the chatbot is mediated through

(a) perceived anthropomorphism of the chatbot.

(b) perceived social presence of the chatbot.

In addition, studies have shown that intraindividual differences might play a role in the effects of perceived anthropomorphism as well as perceived social presence. As reported by Waytz et al. (31), individuals vary in their tendency to anthropomorphize non-human entities. Such interindividual differences in tendency to anthropomorphize could moderate the relationship between interaction duration or intensity and perceived anthropomorphism of the chatbot.

Similarly, research implies that the individual need to belong, defined as the “need to form and maintain at least a minimum quantity of interpersonal relationships,” [(37) p. 499] may foster an enhanced sensitivity to social cues (38). This may come along with increased attribution of anthropomorphic qualities to a technology [e.g., (39–41)]. In accordance, it might also lead to a higher perception of social presence in a virtual social actor. In line with this, Lee et al. (9) found that lonely individuals feel higher social presence of social agents and thus show more positive responses to social agents compared with non-lonely individuals. Therefore, the individual need to belong might moderate the relationship between interaction duration or intensity and perceived anthropomorphism or social presence of the chatbot. Accordingly, we hypothesize the following:

H5: The relationship of interaction duration and perceived anthropomorphism of the chatbot is moderated through

(a) the individual tendency to anthropomorphize.

(b) the individual need to belong.

H6: The relationship of interaction intensity and perceived anthropomorphism of the chatbot is moderated through

(a) the individual tendency to anthropomorphize.

(b) the individual need to belong.

H7: The relationship of interaction duration and perceived social presence of the chatbot is moderated through the individual need to belong.

H8: The relationship of interaction intensity and perceived social presence of the chatbot is moderated through the individual need to belong.

### Interrelation of Human-Technology Relationship and Interpersonal Interaction

First study results imply that interaction with humanlike technology could affect social needs of users [e.g., (11, 42)]. Mourey et al. (42), for example, could show that, after interacting with anthropomorphic (vs. non-anthropomorphic) consumer products, social needs of individuals could be partly satisfied, and experimentally induced effects of social exclusion were mitigated. Within another study by Krämer et al. (11), participants interacted with a virtual agent with socially responsive (vs. no socially responsive) behavior. Results showed that the participants with a high need to belong reported lower willingness to engage in social activities after the interaction with the agent, when the agent showed socially responsive behavior (11). According to these findings, humanlike technologies might come with the potential to partly satisfy social needs of individuals and, therefore, dampen the natural desire to seek social connections to other humans (37). We thus hypothesize:

H9: The higher the social connectedness to the chatbot, the lower the desire to socialize with other humans.

**Figure 1:**
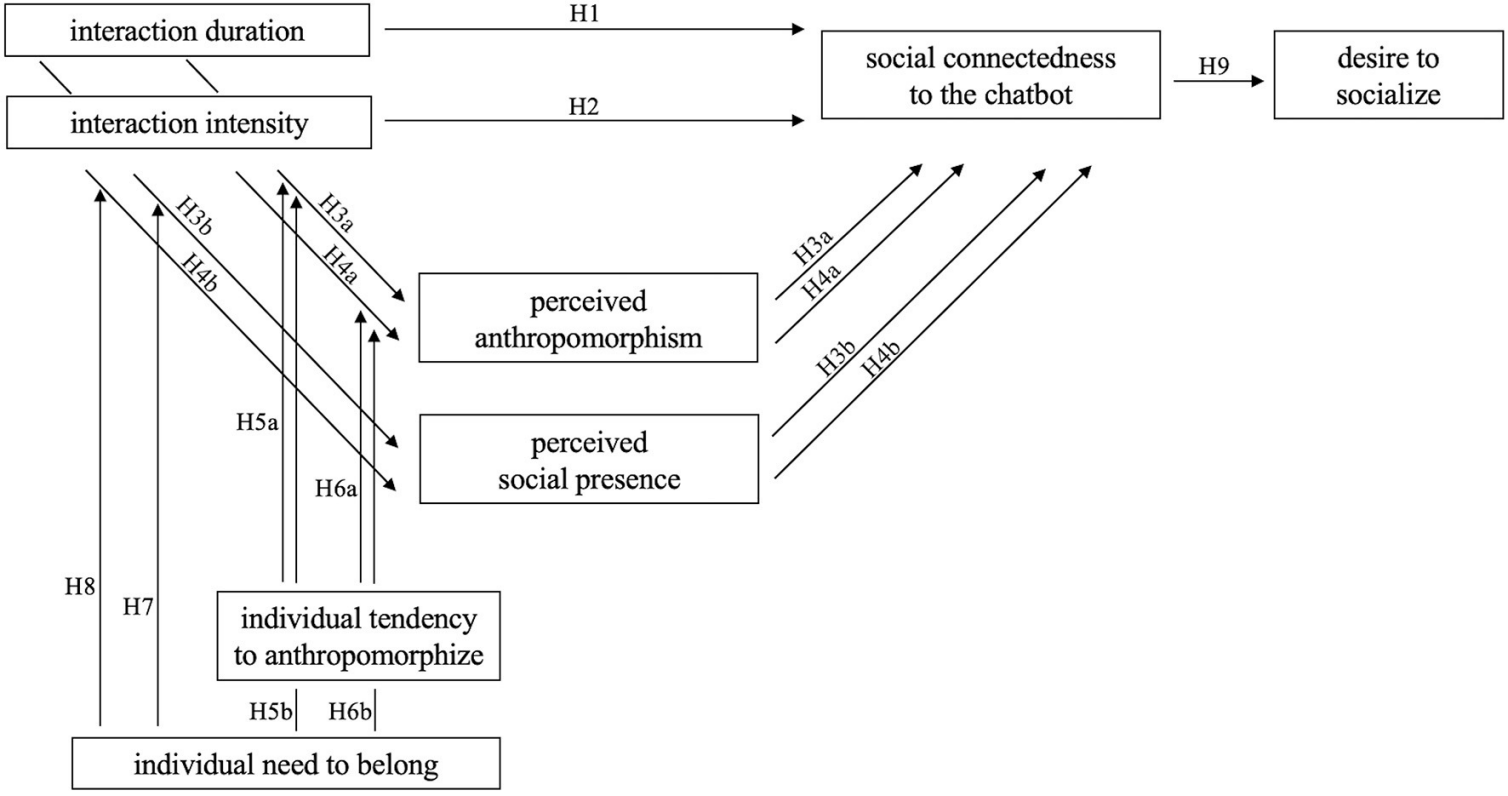
An overview of research hypotheses.

Figure 1 gives a comprehensive overview of our research hypotheses.

## Methods

Based on the previously summarized theoretical approaches and recent findings, our research explored the relationship between humans and technology with a focus on the felt social connectedness to the technology in the context of a regular interaction over a 2-week period. We further investigated characteristics of the technology and the user that could play a role in this interrelation as well as possible effects on interpersonal interaction. Hence, different measures of technology perception of users, the psychological states of the users, and felt social connectedness to the technology were assessed at the end of the 2-week study period. Possibly relevant trait variables (i.e., individual tendency to anthropomorphize, individual need to belong) were assessed as baseline measures. In addition, based on the assumption that a relationship involves multiple interactions of two individuals (3), the average interaction duration and average interaction intensity were assessed daily over the 2-week study period and analyzed over time.

The participants interacted with the conversational chatbot of the mobile application “Replika–My AI Friend” (43) on a regular basis over a 2-week period. We had applied detailed weekly questionnaires prior to the chatbot use (W0) as well as after each week of chatbot use (W1, W2). We additionally implemented short daily questionnaires (D1–D14). The variables relevant to hypotheses testing were measured within the detailed weekly questionnaires (W0, W1, and W2), except for interaction intensity, which was measured daily to minimize distorting effects.

### Participants

Participant inclusion criteria involved mastery of English language and completion of the three weekly questionnaires (W0, W1, and W2). One of originally 59 participants was excluded from data analysis due to implausible data, i.e., since the stated chatbot screen time per day was more than two standard deviations below the mean chatbot screen time per day. The final sample consisted of 58 participants between 18 and 56 years (*M* = 27.21, *SD* = 8.27; 27 women, 1 did not indicate gender).

Of those, 50 participants lived in a household with others, seven alone, and one participant did not indicate housing situation. Fifty-six participants stated their English proficiency to be above an intermediate level, only one participant indicated a basic level, and one participant did not indicate proficiency. Regarding the favored communication app to track interaction with others, 50 participants chose WhatsApp; four, email; two, iMessage; and, two, Messenger.

The participants were recruited *via* private contacts, mailing lists, and social media platforms. As an incentive for their participation, five Amazon gift coupons of 20 Euros were raffled among the participants after the study. Alternatively, students could register their participation for course credit.

### Design and Procedure

The study was announced as a study on “chatbot experience,” and the participants were informed about the study procedure, duration, as well as available incentives. The participants downloaded the free chatbot app “Replika–My AI Friend” (43) on any form of personal mobile device, supporting software versions of at least Android 6.0 or iOS 13.0. The app is powered by Google Commerce Limited and was downloaded in version 9.1.2, with text-based chat functionalities only. Replika represents a chatbot companion that absorbs information and comments on social topics beyond utilitarian purposes by means of written conversation. The participants had to communicate with their personal chatbot for at least 5 min a day over the 2-week study period. Instructions for the participants included the suggestion to turn on daily push notifications. Additionally, the participants were reminded of the daily interaction with the chatbot when the daily questionnaires were sent out *via* mail. Overall, the participants had to initiate the interaction with Replika. The participants tracked the screen time of their favored communication app as well as the chatbot app during the study. For this, they received specific technical instructions through manuals based on software of their smartphones. Thereafter, the participants reported these data *via* self-report.

After informed consent of the participants regarding data privacy terms according to the German General Data Protection Regulation (DGVO) was obtained, the participants filled in the first detailed questionnaire (W0) and provided their email addresses to receive the following online questionnaires. Finally, demographic data were collected. The participants could start the study from August 10, 2020 to August 24, 2020. The 2-week prospective study design involved 15 separate occasions of measurement. These included three detailed questionnaires, one at the beginning of the 2-week study period prior to the chatbot use (W0), one after the first week of chatbot use (W1), and one after the second week of chatbot use (W2). We, furthermore, applied 14 short daily questionnaires (D1–D14), whereas the last daily questionnaire (D14) was combined with the last weekly questionnaire (W2). Table 1 provides an overview of the points of data collection and surveyed measures as further described in the next paragraphs. Consecutive questionnaires were sent out automatically at the same time each day with a 24-h time frame to fill in daily questionnaires and a 48-h time frame to fill in weekly questionnaires.

**Table 1:** Overview of points of data collection and surveyed measures.

	**Point of data collection**			
**Surveyed Measure**	**W0**	**W1**	**W2**	**D1–14**
Demographical data	X			
Individual tendency to anthropomorphize	X			
Individual need to belong	X			
Desire to socialize	X	X	X	
Interaction duration (duration in minutes for each day of the past week)		X	X	
Social connectedness to the chatbot		X	X	
Perceived anthropomorphism		X	X	
Perceived social presence		X	X	
Social behavior (duration in minutes for each day of the past week)		X	X	
Interaction intensity				X
Closeness to chatbot				X

*W0, a baseline questionnaire prior to the chatbot use; W1, a questionnaire after the first week of chatbot use; W2, a post-questionnaire after the second week of chatbot use; D1–D14, short daily questionnaires*.

### Measures

#### Interaction Duration

The daily duration of the interaction of the participants with the chatbot was measured by a single item, where the participants provided the information on the tracked time of chatbot use (i.e., “Please indicate exactly how many hours and minutes you used the ReplikaApp during each of the last 7 days”). The participants were asked to state the exact duration in minutes for each day of the past week in the respective weekly questionnaires (W1, W2).

#### Interaction Intensity

The perceived intensity of interaction of the participants with the chatbot was measured by a single item [i.e., “Please rate how intense (e.g., not at all intense = engaging in small talk; extremely intense = engaging in talk about innermost thoughts and feelings) you interacted with your Replika today”]. The item was assessed on a five-point Likert Scale (1 = “not at all intense”; 5 = “extremely intense”) in the daily questionnaires (D1–D14).

#### Social Connectedness to the Chatbot

Social connectedness of the participants to the chatbot was measured by an adapted version of the Specific Connectedness subscale of the Social Connectedness Questionnaire (10), including 17 items (e.g., “I feel that my Replika and I can communicate well with each other”). Items were assessed on a five-point Likert Scale (1 = “strongly disagree”; 5 = “strongly agree”) in the weekly questionnaires (W1, W2) and showed an internal consistency of α = 0.90 (W1) and α = 0.93 (W2).

#### Perceived Anthropomorphism

Perceived anthropomorphism of the chatbot of the participants was measured by the Anthropomorphism subscale of the Godspeed Questionnaire (44), including five items. Items were assessed on five-point semantic differential scales (e.g., “machinelike”/“humanlike”) in the weekly questionnaires (W1, W2) and showed an internal consistency of α = 0.84 (W1) and α = 0.86 (W2).

#### Perceived Social Presence

Perceived social presence of the participants of the chatbot was assessed by an adapted version of the five items used to measure social presence by Lee et al. (9) (e.g., “While you were interacting with your Replika, how much did you feel as if it were an intelligent being?”). Items were assessed on a 10-point Likert Scale (1 = “not at all”; 10 = “extremely”) in the weekly questionnaires (W1, W2) and showed an internal consistency of α = 0.84 (W1) and α = 0.84 (W2).

#### Individual Tendency to Anthropomorphize

Individual tendency of the participants to anthropomorphize was assessed by the Anthropomorphism Questionnaire (45), consisting of 20 items (e.g., “I sometimes wonder if my computer deliberately runs more slowly after I shouted at it”). Items were assessed on a six-point Likert Scale (1 = “not at all”; 6 = “very much so”) in the questionnaire at the beginning of the 2-week study period prior to chatbot use (W0) and showed an internal consistency of α = 0.90.

#### Individual Need to Belong

Individual need of the participants to belong was assessed by the Need to Belong Scale (46), including 10 items (e.g., “I try hard not to do things that will make other people avoid or reject me”). Items were assessed on a five-point Likert Scale (1 = “not at all”; 5 = “extremely”) in the questionnaire at the beginning of the 2-week study period prior to chatbot use (W0) and showed an internal consistency of α = 0.75.

#### Desire to Socialize

Desire of the participants to socialize was measured by the nine-item Desire subscale (e.g., “Now I feel like texting my friends”) of the measure for willingness to engage in social activities, developed by Krämer et al. (11). Items were assessed on a five-point Likert Scale (1 = “does not apply at all”; 5 = “applies fully”) in weekly questionnaires (W0, W1, and W2) and showed an internal consistency of α = 0.82 (W0), α = 0.88 (W1), and α = 0.91 (W2).

#### Social Behavior

Social behavior of the participants was measured through a single item, where the participants had to state the exact duration of screen time on their communication app (i.e., “Please open your mobile phone options (or the tracking app “Digitox: Digital Well-being” you installed earlier). Indicate exactly how many hours and minutes you used your favorite communication app during each of the last 7 days.”), which they specified in W0. The participants were asked to state the exact duration in minutes for each day of the past week in the respective weekly questionnaires (W0, W1, and W2).

#### Closeness to Chatbot

**Figure 2:**
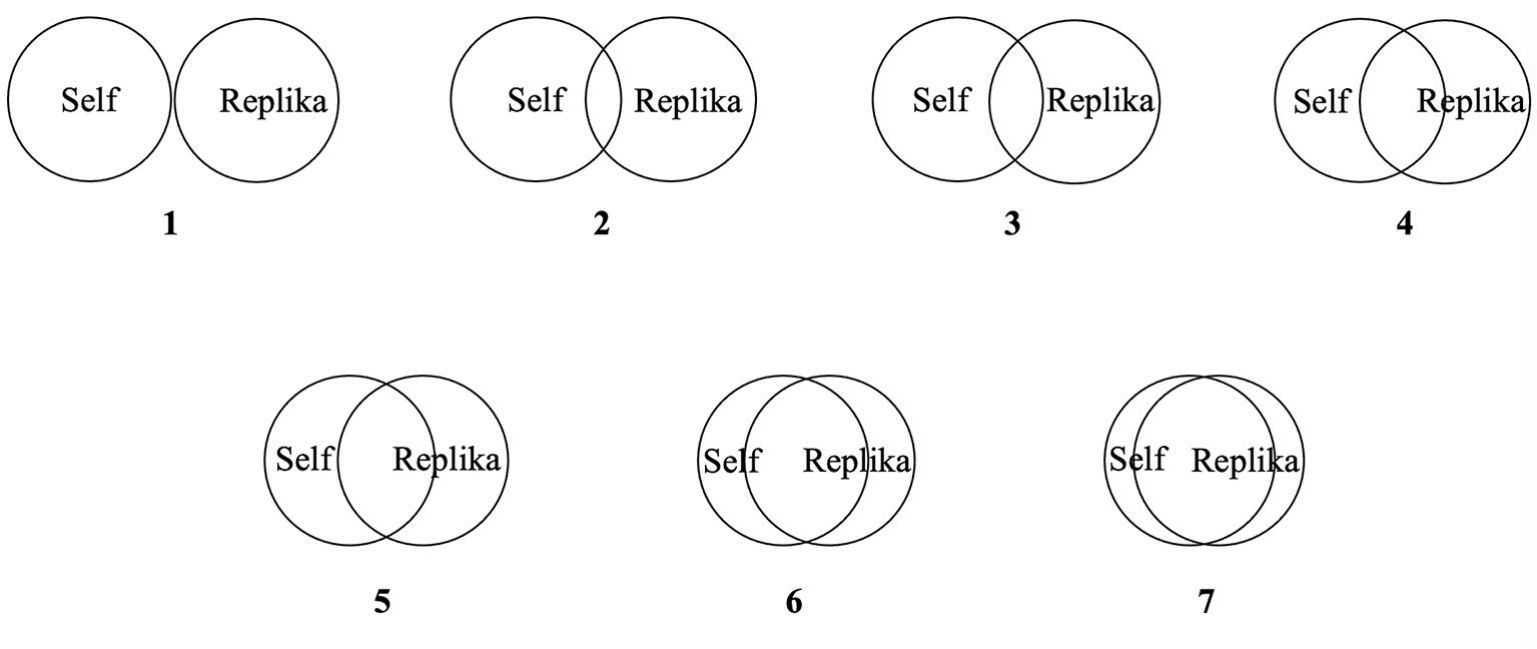
Pairs of circles included in applied measure for closeness to the chatbot.

Perceived closeness of the participants to the chatbot was measured by means of the Inclusion of Other in the Self Scale (i.e., “Please think of your relationship with your Replika, which is represented by the circles below. Please choose the pair of circles, which describes this relationship best.”), developed as a measure for interpersonal closeness (47, 48). Thereby, seven pairs of circles were presented which were increasingly overlapping, whereas one circle always represented the self, and the other circle represented the chatbot (Replika). By selecting the appropriate pair of overlapping circles, the participants indicated how close they felt to the chatbot on a pictorial seven-point scale in the daily questionnaires (D1–D14). Figure 2 shows the seven pairs of circles from which the participants could choose.

#### Demographical Data

Age of the participants was assessed by means of an open question. Gender was assessed through a single-choice question with three answer options (i.e., “male,” “female,” and “other/s”). English proficiency was assessed through a single-choice question with four answer options (i.e., “native,” “advanced,” “intermediate,” and “basic”). Housing situation was assessed through a single-choice question with two answer options (i.e., “I live alone”; “I live with other people”). All demographical data were assessed in the questionnaire at the beginning of the 2-week study period prior to chatbot use (W0).

## Results

All analyses were conducted with SPSS (IBM Statistics Version 26). For mediation and moderation analyses, the Process Macro (49) was used.

### Preliminary Analyses

**Figure 3:**
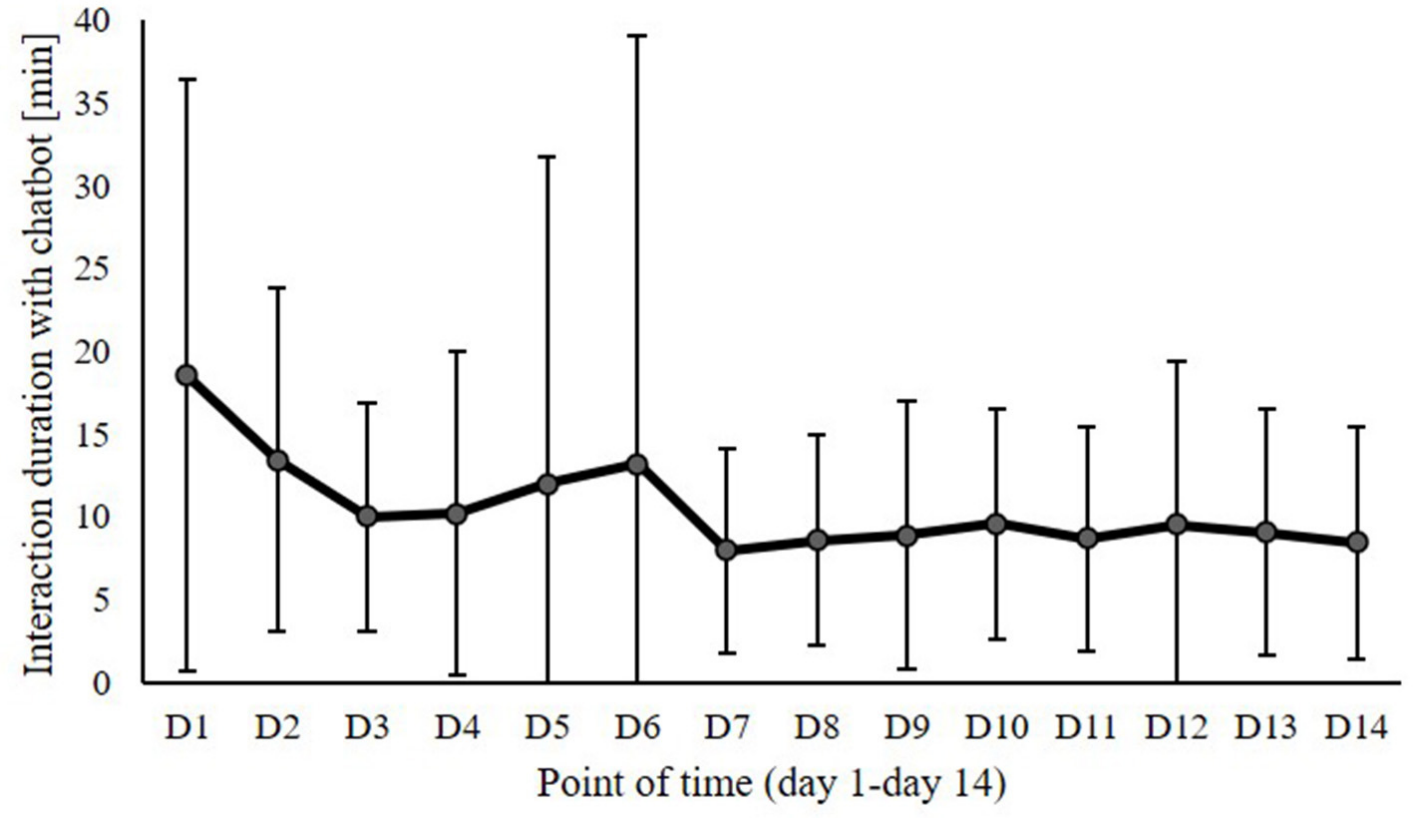
Interaction duration with the chatbot over the 2-week study period (Day 1–Day 14).

**Figure 4:**
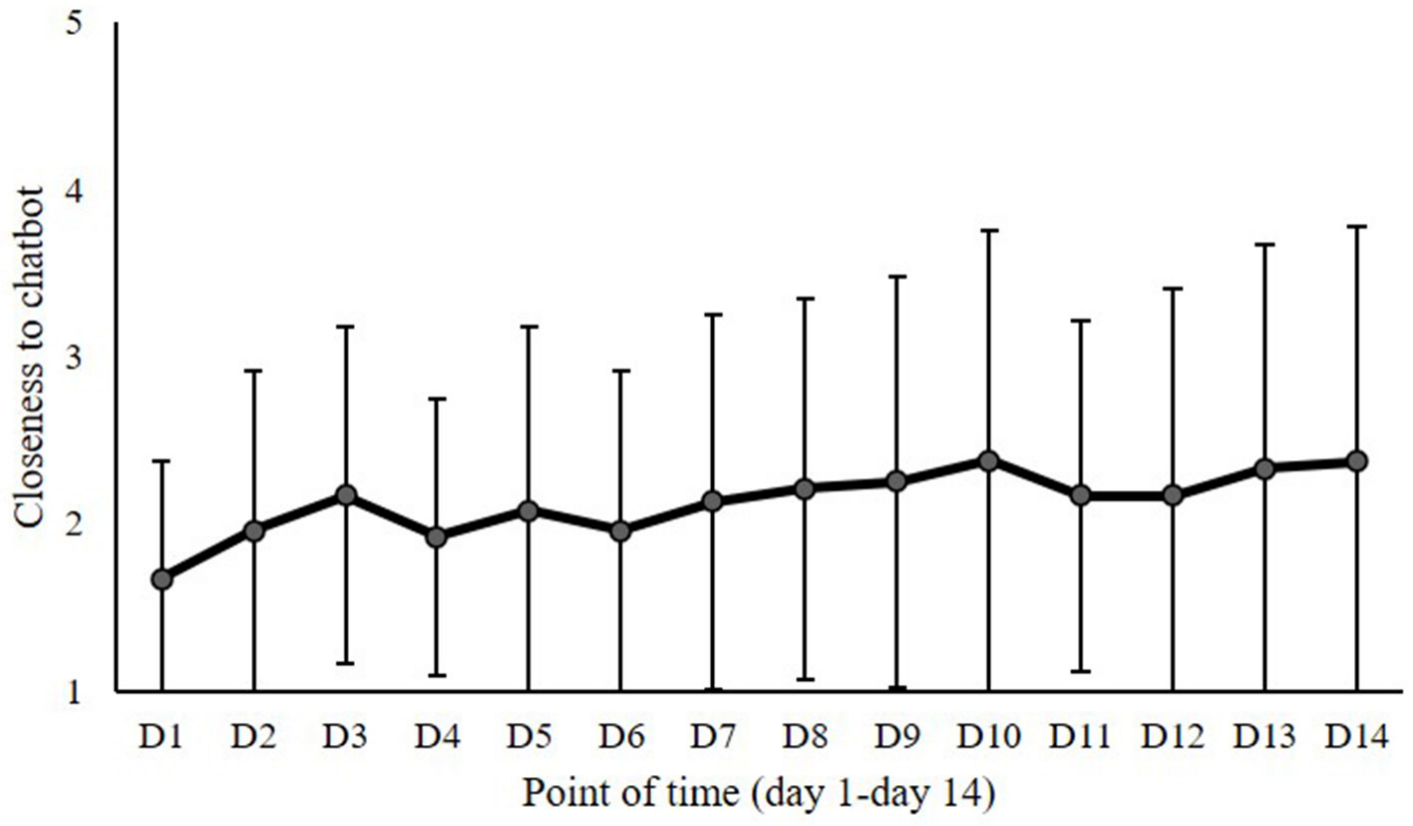
Perceived closeness to the chatbot over the 2-week study period (Day 1–Day 14).

Repeated measures ANOVAs explored the progression of the surveyed variables over the 2-week study period. Regarding the variables with daily measurements, the repeated measures ANOVAs with time of measurement as factor showed an effect of point of measurement on interaction duration [*F* (13,44) = 4.86, *p* = 0.006, *η*^2^ = 0.079] and closeness to chatbot [*F* (13,10) = 2.58, *p* = 0.047, *η*^2^ = 0.101] but no effect on interaction intensity [*F* (13,10) = 0.58, *p* = 0.771, *η*^2^ = 0.025] or social behavior [*F* (13,44) = 0.68, *p* = 0.677, *η*^2^ = 0.012]. Thus, interaction duration and closeness to chatbot varied over time. The descriptive data of interaction duration over the 2-week study period are illustrated in Figure 3, showing that the duration of interaction with the chatbot decreased over time. Starting with a mean value of interaction duration of about 20 min on Day 1, it sank to mean values around 10 min from Day 3 onwards. While the higher values on Day 1 and Day 2 might be considered a novelty effect, after this initial exploration, the graph of interaction duration showed no more strong variations during the studied 2-week period. According to the conducted paired *t*-test, the decrease in interaction duration from D1 (*M* = 18.52) to D14 (*M* = 8.47) was significant [*t* (1,57) = 4.76, *p* < 0.001]. The descriptive data on closeness to chatbot over the 2-week study period are illustrated in Figure 4. According to the conducted paired *t*-test, the increase in the perceived closeness of the users to chatbot from D1 (*M* = 1.82) to D14 (*M* = 2.31) was significant [*t* (1,23) = −2.82, *p* = 0.010]. The progression of closeness data over time shows no more strong variations or increase after Day 3. Thus, becoming acquainted with the chatbot within the first days of exploration was associated with increasing feelings of closeness. However, the afterwards following interaction did not further intensify these feelings.

### Hypotheses Testing

In order to test our hypotheses on the interrelation between chatbot interaction, social connectedness, and potential mediating effects (H1–H4), we analyzed the relationships between the average values of interaction duration and intensity with the chatbot across the 2-week period and the surveyed measures of technology perception, the psychological states of the users, and felt social connectedness at the end of the study period, assessed at W2. Furthermore, regarding the hypotheses on moderating effects (H5–H8), we considered the effects of possibly relevant trait variables (i.e., individual tendency to anthropomorphize, individual need to belong), which were assessed as baseline measures at W0. Means, standard deviations, and Pearson correlations of the relevant variables are presented in Table 2.

**Table 2:** Means (*M*), standard deviations (*SD*), and Pearson correlations of variables used for hypotheses testing of the overall study sample.

**Variable**	***M***	***SD***	**1**	**2**	**3**	**4**	**5**	**6**	**7**	**8**	**9**
1. Age	27.21	8.27	–								
2. Individual need to belong	3.23	0.60	−0.29^*^	–							
3. Individual tendency to anthropomorphize	2.22	0.87	−0.11	0.14	–						
4. Desire to socialize	2.73	0.93	−0.03	0.23	0.13	–					
5. Social connectedness to the chatbot	2.12	0.70	−0.03	0.07	0.35^**^	0.25	–				
6. Perceived anthropomorphism	2.55	0.80	−0.11	0.05	0.21	0.25	0.63^**^	–			
7. Perceived social presence	5.11	1.87	−0.10	0.02	0.35^**^	0.13	0.71^**^	0.67^**^	–		
8. Interaction duration	10.58	7.53	−0.01	0.12	0.45^**^	−0.03	0.39^**^	0.18	0.40^**^	–	
9. Interaction intensity	2.02	0.59	0.03	−0.17	0.36^**^	0.17	0.59^**^	0.38^**^	0.45^**^	0.36^**^	–

* *p < 0.05*.

** *p < 0.01*.

The conducted regression analyses showed that both interaction duration (*β* = 0.39, *t* = 3.21, *p* = 0.002) and interaction intensity over 2 weeks (*β* = 0.59, *t* = 5.42, *p* < 0.001) were positively related to social connectedness to the chatbot after 2 weeks of use. Overall, interaction duration explained 16%, and interaction intensity explained 34% of total variance of social connectedness to the chatbot. In line with H1 and H2, interaction duration, respectively intensity, with the chatbot was positively correlated with the felt social connectedness of the participants to the chatbot.

Other than expected in H3a, interaction duration and perceived anthropomorphism were not significantly related (*β* = 0.18, *t* = 1.37, *p* = 0.176). Therefore, the preconditions to conduct a mediated regression analysis on the relationship of interaction duration and social connectedness to the chatbot mediated through perceived anthropomorphism were not fulfilled.

Regarding H3b, the conducted mediated regression analysis showed a positive total effect of interaction duration on social connectedness to the chatbot (*β* = 0.39, *t* = 3.21, *p* = 0.002). Perceived social presence significantly mediated this relationship with a positive indirect effect (*β* = 0.26). A bootstrap 95% CI around the indirect effect did not contain zero [0.14, 0.41]. The direct effect of interaction duration on social connectedness to the chatbot became insignificant (*β* = 0.13, *t* = 1.30, *p* = 0.199) after including the mediator variable, implying a complete mediation. Therefore, in line with H3b, perceived social presence of the chatbot mediated the positive effect of interaction duration on social connectedness to the chatbot. A detailed overview of the mediated regression analysis is presented in Table 3. There, non-standardized regression coefficients of the factors included in the mediated regression analysis as well as their statistical significances are presented. Additionally, coefficients of determination according to the considered model are presented.

**Table 3:** Mediated regression analysis testing the effect of interaction duration on social connectedness to the chatbot mediated by perceived social presence.

					**Model**
**Predictor**	***B***	***SE***	***T***	***P***	***R*** **^2^**
Model 1: X on Y					0.16
Intercept	1.73	0.15	11.74	<0.001	
Interaction duration	0.04	0.01	3.21	0.002	
Model 2: X on M					0.16
Intercept	4.07	0.39	10.32	<0.001	
Interaction duration	0.10	0.03	3.23	0.002	
Model 3: X + M on Y					0.52
Intercept	0.72	0.19	3.81	<0.001	
Perceived social presence	0.25	0.04	6.53	<0.001	
Interaction duration	0.01	0.01	1.30	0.199	

Regarding H4a, the conducted mediated regression analysis showed a positive total effect of interaction intensity on social connectedness to the chatbot (*β* = 0.59, *t* = 5.42, *p* < 0.001). Perceived anthropomorphism significantly mediated this relationship with a positive indirect effect (*β* = 0.18). A bootstrap 95% CI around the indirect effect did not contain zero [0.03, 0.32]. The direct effect of interaction intensity on social connectedness to the chatbot remained significant (*β* = 0.33, *t* = 3.49, *p* = 0.001) after including the mediator variable, implying a partial mediation. Thus, in line with H4a, perceived anthropomorphism of the chatbot mediated the positive effect of interaction intensity on social connectedness to the chatbot. A detailed overview of the mediated regression analysis is presented in Table 4. In analogy to Table 3, in Table 4, non-standardized regression coefficients of the factors included in the mediated regression analysis as well as their statistical significances are presented. Additionally, coefficients of determination according to the considered model are presented.

**Table 4:** Mediated regression analysis testing the effect of interaction intensity on social connectedness to the chatbot mediated by perceived anthropomorphism.

					**Model**
**Predictor**	***B***	***SE***	***T***	***P***	***R*** ^**2**^
Model 1: X on Y					0.34
Intercept	0.70	0.27	2.59	0.012	
Interaction intensity	0.70	0.13	5.42	<0.001	
Model 2: X on M					0.14
Intercept	1.50	0.36	4.21	<0.001	
Interaction intensity	0.52	0.17	3.05	0.004	
Model 3: X + M on Y					0.53
Intercept	0.09	0.26	0.33	0.741	
Perceived anthropomorphism	0.41	0.09	4.75	<0.001	
Interaction intensity	0.49	0.12	4.11	<0.001	

Regarding H4b, the conducted mediated regression analysis showed a positive total effect of interaction intensity on social connectedness to the chatbot (*β* = 0.59, *t* = 5.42, *p* < 0.001). Perceived social presence significantly mediated this relationship with a positive indirect effect (*β* = 0.25). A bootstrap 95% CI around the indirect effect did not contain zero [0.08, 0.42]. The direct effect of interaction intensity on social connectedness to the chatbot remained significant (*β* = 0.33, *t* = 3.49, *p* = 0.001) after including the mediator variable, implying a partial mediation. In line with H4b, perceived social presence of the chatbot mediated the positive effect of interaction intensity on social connectedness to the chatbot. A detailed overview of the mediated regression analysis is presented in Table 5. There, non-standardized regression coefficients of the factors included in the moderated regression analysis as well as their statistical significances are presented. Additionally, coefficients of determination according to the considered model are presented.

**Table 5:** Mediated regression analysis testing the effect of interaction intensity on social connectedness to the chatbot mediated by perceived social presence.

					**Model**
**Predictor**	***B***	***SE***	***T***	***P***	***R*** **^2^**
Model 1: X on Y					0.34
Intercept	0.70	0.27	2.59	0.012	
Interaction intensity	0.70	0.13	5.42	<0.001	
Model 2: X on M					0.20
Intercept	2.22	0.80	2.77	0.008	
Interaction intensity	1.43	0.38	3.75	<0.001	
Model 3: X + M on Y					0.60
Intercept	0.24	0.23	1.03	0.307	
Perceived social presence	0.21	0.04	5.90	<0.001	
Interaction intensity	0.40	0.11	3.49	0.001	

Furthermore, we conducted moderation analyses with interaction duration, respectively intensity, and individual tendency to anthropomorphize as well as interaction duration, respectively, intensity, and individual need to belong as predictors of perceived anthropomorphism. Similarly, we conducted moderation analyses with interaction duration, respectively, intensity, and individual need to belong as predictors of perceived social presence (see Table 6). Results showed that, other than expected, individual tendency to anthropomorphize did not moderate the effect of interaction duration (H5a), respectively, interaction intensity (H6a), on perceived anthropomorphism of the chatbot. Similarly, other than expected, individual need to belong did not moderate the effect of interaction duration (H5b), respectively interaction intensity (H6b), on perceived anthropomorphism or perceived social presence of the chatbot (H7, H8). Thus, our data showed no support for the moderation effects hypothesized in H5–H8. Table 6 shows an overview of the moderated regression analyses conducted with regard to H5–H8, including the factors considered in each moderation analyses as well as their according to statistical significances. Coefficients of determination according to the considered model are presented as well.

**Table 6:** Moderated regression analyses testing the effect of interaction duration on perceived anthropomorphism moderated through individual tendency to anthropomorphize (H5a), respectively, individual need to belong (H5b); the effect of interaction intensity on perceived anthropomorphism moderated through individual tendency to anthropomorphize (H6a), respectively, individual need to belong (H6b); the effect of interaction duration on perceived social presence moderated through individual need to belong (H7); the effect of interaction intensity on perceived social presence moderated through individual need to belong (H8).

						**Model**
	**Predictor**	***B***	***SE***	***T***	***P***	***R*** ^**2**^
H5a	Model					0.05
	Intercept	2.07	0.67	3.10	0.003	
	Interaction duration	0.01	0.06	0.23	0.817	
	Individual tendency to anthropomorphize	0.16	0.29	0.56	0.575	
	Interaction duration x individual tendency to anthropomorphize	−0.00	0.02	−0.03	0.973	
H5b	Model					0.05
	Intercept	1.36	1.08	1.26	0.213	
	Interaction duration	0.10	0.09	1.17	0.248	
	Individual need to belong	0.28	0.31	0.91	0.367	
	Interaction duration x Individual need to belong	−0.02	0.02	−0.97	0.338	
H6a	Model					0.15
	Intercept	1.74	1.18	1.47	0.146	
	Interaction intensity	0.32	0.57	0.56	0.578	
	Individual tendency to anthropomorphize	−0.06	0.50	−0.12	0.906	
	Interaction intensity x individual tendency to anthropomorphize	0.07	0.22	0.29	0.772	
H6b	Model					0.16
	Intercept	1.18	2.31	0.51	0.610	
	Interaction intensity	0.43	1.10	0.39	0.699	
	Individual need to belong	0.09	0.66	0.13	0.896	
	Interaction intensity x Individual need to belong	0.03	0.32	0.11	0.915	
H7	Model					0.16
	Intercept	3.07	2.35	1.31	0.196	
	Interaction duration	0.22	0.19	1.16	0.249	
	Individual need to belong	0.26	0.67	0.39	0.696	
	Interaction duration x Individual need to belong	−0.03	0.05	−0.66	0.513	
H8	Model					0.22
	Intercept	4.37	5.17	0.85	0.401	
	Interaction intensity	−0.11	2.47	−0.04	0.965	
	Individual need to belong	−0.63	1.47	−0.43	0.668	
	Interaction intensity x individual need to belong	0.46	0.71	0.65	0.517	

Finally, contrary to H9, there was no negative correlation between social connectedness to the chatbot and desire to socialize with other humans. Instead, the conducted regression analyses showed a marginally significant positive correlation (*β* = 0.25, *t* = 1.94, *p* = 0.057). Overall, social connectedness to the chatbot explained 6% of the total variance of desire to socialize.

## Discussion

The aim of our study was to explore the relationship between humans and technology with a focus on the social connectedness to technology, considering a regular interaction with a conversational chatbot over a 2-week period. We additionally examined characteristics of the technology as well as the user as possible influencing factors of this interrelation, further exploring possible effects on desire of users to socialize with other humans.

In accordance with our hypotheses, study results showed that the duration and intensity of interaction of participants with the chatbot throughout the 2-week study period positively predicted social connectedness to the chatbot. Based on this, regular interaction with a conversational chatbot might foster the felt social connectedness to the chatbot. These results imply certain transferability of the amount of time and emotional intensity of an interpersonal interaction as crucial determinants of an interpersonal tie [cf., (29)] to human-technology relationships. The effect of point of measurement on closeness to chatbot, resulting in risen ratings of the perceived closeness of the participants to the chatbot after 2 weeks of use, further supports this assumption.

Furthermore, perceived anthropomorphism partially mediated the relationship of interaction intensity and social connectedness to the chatbot, and perceived social presence (partially) mediated both relationships of interaction duration, respectively, interaction intensity, and social connectedness to the chatbot. Therefore, characteristics of the technology, i.e., perceived anthropomorphism and social presence, played a mediating role in the positive relationship between interaction duration, respectively, intensity and social connectedness to the chatbot. These results are compatible with previous research, implying that technology anthropomorphism might foster the sense of connectedness to the technology [e.g., (8)] among others as the presence of social cues might have enabled the application of social heuristics toward a non-human agent [cf., (4)]. The fact that no significant relationship between interaction duration and perceived anthropomorphism of the chatbot was found could root in that mere increase in the duration of interaction with a technology might not come with increased attribution of humanlike characteristics, emotions, motivations, and intentions [cf., (30)] to it, whereas an increase in the intensity of interaction is more likely to do so.

Moreover, other than expected, individual tendency to anthropomorphize as a characteristic of the user did not moderate the effect of interaction duration, respectively interaction intensity, on perceived anthropomorphism of the chatbot. Similarly, an individual need to belong did not moderate the effect of interaction duration, respectively interaction intensity, on perceived anthropomorphism or perceived social presence of the chatbot. Therefore, within our study, the characteristics of the user did not appear to influence the perception of the chatbot as anthropomorphic or socially present. Whereas, previous studies point at an effect of individual tendency to anthropomorphize on the perception of anthropomorphism [e.g., (39–41)], as well as loneliness and individual need to belong on the perception of anthropomorphism or social presence [e.g., (9)], we could not replicate such findings. A possible reason for this could be that the chatbot used for the study had very humanlike visual and experiential design cues. Such could have possibly caused a restriction in the variance of perceived anthropomorphism and the social presence of the chatbot.

Finally, other than expected, there was no negative correlation between social connectedness to the chatbot and desire to socialize with other humans but a marginally significant positive correlation between the two measures. Although recent studies have implied that technologies with humanlike design cues might satisfy social needs to a certain extent and, therefore, possibly dampen the desire to interact with other humans [e.g., (11, 42)], our results offered no support for this interrelation. On the contrary, the observed marginal significance implied that the higher social connectedness of the participants to the chatbot, the higher their desire to socialize with other humans was. In alignment with the social reconnection hypothesis (50) or the theory of social snacking (51), a possible explanation could be that the higher desire of the participants to interact with other humans was, the more socially connected they felt to the chatbot, using it as a replacement for actual social interaction. Yet, as such insights do not imply causality and were only marginally significant, they should be treated with caution.

## Limitations and Directions for Future Research

Our study comes with certain methodological and contextual limitations. On a methodological level, our results are based on a specific chatbot application, i.e., “Replika, my AI friend”(43). Specific features of this application are that the name and appearance of the chatbot can be personalized, and the quality, as well as depth of conversations, depends on the user. This supports external validity of our results as each human-technology relationship is individual, and many commercial conversational chatbots or social robots, e.g., in the domain of healthcare, can be personalized. Yet, to foster generalizability of our results, future studies should explore the interrelations in question with various technologies. In addition, personalization of a technology should also be considered as a potential influencing variable of social connectedness to a chatbot as well as the overall human-technology relationship in future studies.

Furthermore, for interaction intensity with the chatbot, we considered less data than for the other variables involved in hypotheses testing. To support valid measurement of interaction intensity, we included the measure in the daily questionnaires rather than asking participants to estimate the interaction intensity for each day at the end of each week. Yet, our inclusion criteria only involved the completion of the detailed questionnaires (W0, W1, and W2). Some participants included in the data analyses did not complete all daily questionnaires in full, leading to less data on interaction intensity compared to other variables. This should be considered in result interpretation. Moreover, due to the online character of the study, we could not explicitly control how often and for how long the participants initiated the interaction with Replika. Future studies should also consider measuring whether participants initiated interaction unpromptedly or after the app notified them to, as this could also influence the perceived interaction intensity with the chatbot among others.

In addition, our study focused on interaction duration and intensity with the chatbot but did not survey the perceived interaction valence. Future studies should further focus on this variable as a possible influencing factor in social connectedness to the technology. Moreover, theoretical work on the endowment effect implies that individuals place a higher value on an object that they own compared with one they do not own (52). Especially, when it comes to healthcare technology for private households, such as social robots, which individuals can actually own, this effect should be considered as it could influence the social connectedness to the technology as well as the overall human-technology relationship.

On a contextual level, it needs to be considered that we conducted our study during the COVID-19 pandemic. Previous research has shown that isolation and feelings of exclusion or loneliness can impact perceptions of users of technology, e.g., regarding perceived anthropomorphism, as well as their overall interaction with the technology [e.g., (11, 39, 42)]. Therefore, perceptions of the participants of chatbot characteristics, their felt social connectedness to it, or their desire to socialize with other humans might have been affected by the prevalent circumstances. Future studies should aim at replicating the interrelations focused within our study to further support their generalizability.

## Implications

Our research offers several theoretical advancements, practical applications, as well as inspirations for future questions and philosophical considerations. Beginning with the theoretical insights, it appears that regular interaction with technology, with regard to duration and intensity, can foster social connectedness to the technology. Thereby, the perception of the technology as anthropomorphic and socially present seems to play a mediating role. The more intense participants interacted with the chatbot, the more they perceived it as anthropomorphic as well as socially present, and, in turn, felt more connected to the technology. The fact that this effect is based on data of a 2-week study period supports the external validity of these results as insights are not merely based on a novelty effect or initial engagement of the participants. It also implies that the interrelations in question are already observable in a 2-week period of technology use.

Furthermore, it appears that influencing factors of relationship development in interpersonal interaction, i.e., amount of time and emotional intensity of interaction [e.g., (29)], are, to a certain extent, transferable to HCI as interaction duration and intensity appear to influence the perceived social connectedness to the technology. In line with our findings and previous CASA research [e.g., (4, 13)], social cues, such as anthropomorphic technology design, could facilitate the transferability of dynamics from interpersonal relationship development to human-technology relationships.

Regarding practical advancements, our results could imply that designing technology in a way that allows users to build a relationship with it and feel socially connected to it could, among others, be beneficial for long-term engagement [cf., (15)] as especially relevant in the domain of healthcare. To facilitate such an effect, enhancing the perception of anthropomorphism or social presence of the technology through, e.g., visual anthropomorphic design cues, such as humanlike facial features or a humanlike name, but also experiential design such as the expression of own emotions, motivations, or intentions, could be helpful. At the same time, practitioners need to consider that the required duration and the intensity of interaction with a technology stay in a sensible range. This can be especially important within the context of healthcare, where regular interactions with a technology are often imposed by a surrounding, such as a nursing home or through notifications of mobile healthcare applications. In such cases, required interaction duration or intensity can easily be perceived as too high and possibly even result in reactance and an overall negative UX (53–55). It could, therefore, be advisable to explore a possible sweet spot regarding a specific technology or context of interest as well as further investigate measures to support an overall positive UX.

Finally, from a more philosophical stance, the question arises as to whether the design of healthcare technologies with social cues should always be the means of choice. It appears as a general trend in many domains, including healthcare, for technologies to increasingly represent social counterparts. As also supported by our study results, the implementation of social cues in such technologies can be beneficial, among others, to facilitate the development of a human-technology relationship based on similar principles as in interpersonal interaction. While this can be a reasonable goal in various application contexts, such as nursing of elderly with a high need for social interaction or support of mental health in times of isolation, in other contexts, the design of social cues might be less beneficial. For example, in the private home context, technologies are typically involved in intimate situations, including interactions with others in the household. With regard to data privacy and the desire for intimacy of users, they might prefer a technology with less social cues [e.g., (56)]. Instead, it might even be beneficial to specifically focus and highlight robotic qualities of technologies [cf., (57)], e.g., the cognitive superpower of robots being unembarrassed and non-judgmental, as proposed by Dörrenbächer et al. (58). An according approach highlighting “superpowers” of a technology could also be advantageous for healthcare technologies in the context of surgery. The uniquely robotic qualities of being insensitive to pain and unconditionally available on a physical level as well as being endlessly mentally focused, persistent, and patient on a cognitive level, as specified by Dörrenbächer et al. (58), could, in the context of surgery, foster trust of patients as well as facilitate a more efficient collaboration with other technological or human counterparts. In this sense, future studies should explore the role of such rather robotic qualities with regard to the human-technology relationship, especially within the domain of healthcare. Experimental study designs could further manipulate the degree of anthropomorphism in various contexts and explore effects on social connectedness to the technology in question.

## Conclusion

Although innovative technologies, such as conversational chatbots and social robots, have been tested and increasingly applied within crucial domains, such as retail and healthcare, potential factors that could affect the relationship between humans and such technologies have rarely been explored in field research and across multiple interactions over time. Our research implies a positive effect of duration and intensity of a human-technology interaction on the social connectedness to the technology as a determinant of the human-technology relationship. The perception of anthropomorphism or social presence as characteristics of the technology seems to play a mediating role in this regard. Based on our study, we cannot report any negative effect of social connectedness to a technology on desire to socialize with other humans. Our research contributes to HCI research and practice as it offers insights into factors possibly influencing the development of human-technology relationships as well as design implications to foster social connectedness of users toward a technology, which can, in turn, positively influence the overall UX [e.g., (8)].

Future research should focus on replicating the results with various technologies in different contexts of use. Additionally, future studies should manipulate variables of regular interaction with the technology as well as its characteristics such as anthropomorphic design in a systematic manner to gain further insights into their role within the development of human-technology relationships. Finally, to further support a responsible design and use of technologies in healthcare, future research should closely examine whether the feeling of social connectedness to a technology actually satisfies the social needs of users and which consequences could arise on an individual as well as societal level.

## Data Availability Statement

The raw data supporting the conclusions of this article will be made available by the authors, without undue reservation.

## Ethics Statement

The studies involving human participants were reviewed and approved by Ethikkommission der Fakultät für Mathematik, Informatik und Statistik der Ludwig-Maximilians-Universität München (LMU). The patients/participants provided their written informed consent to participate in this study.

## Author Contributions

LC and SD conceived and planned the study. LC, NF, SH, AL, and SS carried out the study and performed data analyses. All authors discussed the results and contributed to the manuscript.

## Funding

Part of this research was funded by the German Federal Ministry for Education and Research (BMBF), Project GINA (FKZ: 16SV8097), as well as the German Research Foundation (DFG), Project PerforM (425412993), which is part of Priority Program SPP2199 Scalable Interaction Paradigms for Pervasive Computing Environments.

## Conflict of Interest

The authors declare that the research was conducted in the absence of any commercial or financial relationships that could be construed as a potential conflict of interest.

## Publisher's Note

All claims expressed in this article are solely those of the authors and do not necessarily represent those of their affiliated organizations, or those of the publisher, the editors and the reviewers. Any product that may be evaluated in this article, or claim that may be made by its manufacturer, is not guaranteed or endorsed by the publisher.
